# Exposure to protracted low-dose ionizing radiation and incident dementia in a cohort of Ontario nuclear power plant workers

**DOI:** 10.5271/sjweh.4246

**Published:** 2025-11-01

**Authors:** Brianna Frangione, Ian Colman, Franco Momoli, Estelle Davesne, Robert Talarico, Chengchun Yu, Paul J Villeneuve

**Affiliations:** 1 Department of Neuroscience, Carleton University, Ottawa, Canada.; 2 School of Epidemiology and Public Health, University of Ottawa, Ottawa, Canada.; 3 Nuclear Safety and Radiation Protection Authority (ASNR), Fontenay-aux-Roses, France.; 4 ICES uOttawa (Formerly known as Institute for Clinical Evaluative Sciences), Ottawa, Canada.

**Keywords:** dementia incidence, occupational health, radiation risk

## Abstract

**Objectives:**

Emerging evidence suggests that low-dose ionizing radiation increases the risk of neurodegenerative diseases. Past studies have relied on death data to identify dementia, and these are prone to under-ascertainment and complicate the estimation of health risks as individuals tend to live with dementia for many years following onset. We present findings from the first occupational cohort to investigate dementia risk from low-dose radiation using incident outcomes.

**Methods:**

This is a retrospective cohort of 60 874 Ontario Nuclear Power Plant workers from the Canadian National Dose Registry. Personal identifiers were linked to Ontario population-based administrative health data. Incident dementias between 1996 and 2022 were identified using a validated algorithm based on physician, hospital, and prescription drug data. Individual-level annual estimates of whole-body external ionizing radiation were derived from personal workplace monitoring. The incidence of dementia among these workers was compared to a random sample of Ontario residents matched by sex, age, and residential area. Internal cohort analysis using Poisson and linear excess relative risk (ERR) models, adjusted for sex, attained age, calendar period, and neighborhood income quintile, were used to characterize the shape of the exposure-response curve between low-dose cumulative radiation (lagged 10 years) and incident dementia.

**Results:**

There were 476 incident dementias and 867 028 person-years of follow-up. The mean whole-body lifetime accumulated exposure at the end of follow-up was 11.7 millisieverts (mSv). Workers with cumulative exposure between 50–100 mSv had an increased risk of dementia [RR 1.50, 95% confidence interval (CI) 0.99–2.28] compared to those unexposed. Spline analysis suggested that the dose–response relationship was non-linear. The linear ERR per 100 mSv increase in exposure was 0.704 (95% CI 0.018–1.390).

**Discussion:**

Our findings suggest that low-dose exposure to ionizing radiation increases the risk of incident dementia.

There is an important need to understand the health effects of ionizing radiation, as exposure, to some degree, is ubiquitous. Millions worldwide are employed in occupations where exposures are several orders of magnitude higher than background levels, highlighting the importance of assessing and mitigating potential health risks (1). Studies on these workers can help inform risks not only in occupational settings, but also in the general population. Indeed, exposure to low-dose ionizing radiation [cumulative doses <100 millisieverts (mSv)] has been the focus of a number of occupational cohorts (2). Several studies have been conducted on workers exposed to chronic low-dose radiation in the nuclear industry, but almost all studies have assessed the effects on cancer incidence and mortality (2). There is emerging evidence of other adverse health effects of low-dose ionizing radiation, including cardiovascular diseases (3), adverse birth outcomes (4), glaucoma (5), and cataracts (6).

Recent work has implicated ionizing radiation as a risk factor for neurodegenerative diseases, such as dementia (7). There are several possible pathways whereby ionizing radiation increases the risk of dementia. It is an established factor for premature aging (7) via the production of reactive oxygen species and oxidative stress (8). As both high and low doses of radiation can alter brain cell and cognitive functions (8) and affect neurological integrity, any level of exposure may contribute to the development of dementia (9).

A recent systematic review and meta-analysis of exposure to ionizing radiation and dementia identified eight studies with a summary increased risk of dementia among those exposed to radiation of 1.11 [95% confidence interval (CI) 1.04–1.18] per increase of 100 mSv (10). With one exception, all existing studies (11–21) of radiation exposure and dementia have identified outcomes using death registration data, often by using broadly-based disease categories that have included other neurological conditions. The findings from these studies have been mixed, with some reporting increased mortality risks (14, 17), others showing decreased risks (15, 16, 19), and some reporting null associations (11–13, 21). Of the studies that focused specifically on dementia mortality, the findings have similarly been inconsistent with increased (14, 18, 20), decreased (12, 15, 16), or no statistically significant associations reported (11, 21). For the single study of incident dementia, Yamada et al (22) found a non-statistically significant reduced risk of dementia from exposure to radiation among Japanese Atomic Bomb Survivors. However, these findings are somewhat limited as the study was constrained by a small sample size of 2 286 participants, and the findings are not directly comparable to health risks from protracted low doses occurring over many years.

In our view, the reliance on death registrations to identify dementia outcomes is a critical shortcoming of past research. Misclassification of outcome is prone to occur as death certificates miss a large portion of those who were living with dementia (23). This poses a substantial threat to validity, as evidenced by recent work by Wachterman et al (23), who found that nearly 40% of deaths among US residents with dementia in a long-term care facility were not recorded. Elsewhere, a US-based study estimated that the number of dementia deaths was approximately three times higher than rates derived from death registrations alone (24). We also note that for etiological studies, the analysis of dementia incidence is preferred over mortality because incidence can better provide insight into the latency interval between exposure and disease onset. This is particularly the case for an outcome like dementia, where individuals often live with the condition for a long time (25). On average, individuals live approximately 4–8 years after being diagnosed with Alzheimer’s disease, but some can live as long as 20 years, depending on various factors (25). Finally, epidemiological studies that use dementia incidence rather than mortality-based measures benefit from a greater number of outcomes. This can produce non-trivial gains in statistical precision, particularly for relatively small occupational cohorts. For all the above reasons, larger-scale longitudinal studies that capture incident disease are better suited to providing insights into causal associations between exposure and dementia.

The Canadian National Dose Registry (NDR) has collected data from nearly one million radiation-exposed workers since the 1950s (26). These data have been used in several studies that investigated the health impacts of occupational exposure to ionizing radiation (3, 27). However, it has primarily been used to study the radiation health effects for mortality and cancer incidence. To date, it has not been used to identify other incident outcomes apart from our recent study examining the risks of cataract surgeries and glaucoma among Ontario nuclear power plant (NPP) workers (6). To our knowledge, the analysis presented herein represents the first attempt to characterize the risk of incident dementia in relation to protracted occupational exposure to ionizing radiation.

## Methods

### The Canadian National Dose Registry

The Canadian NDR is owned and operated by Health Canada. It contains dose records of Canadian workers monitored for occupational exposure to ionizing radiation and dates back to the 1950s (26). The Nuclear Safety and Control Act provides the Canadian Nuclear Safety Commission (CNSC) authority to regulate the development, production, and use of nuclear energy and the production, possession, and use of nuclear substances, prescribed equipment, and prescribed information. As part of these regulations, the CNSC requires those licensed to use nuclear energy control and ascertain doses to workers. These doses must be monitored by a licensed dosimetry service when workers have a reasonable probability of receiving an effective annual dose of at least five mSv. The NDR captures several types of radiation exposure, including external gamma, beta, x-rays, and neutrons, as well as internal radionuclides such as tritium and radon (26).

### Occupational radiation exposure

For each worker in the NDR, annual summary doses for whole-body external and internal radiation are collected from each organization where they were monitored. The external doses are penetrating (whole-body) gamma doses, expressed as effective doses in mSv, assessed through the dose equivalent Hp[10]. In this study, the whole-body dose estimates also included the contribution from tritium and other less common radionuclides (28). A complete list of the radiation types included in the overall dose calculation is provided in supplementary material (www.sjweh.fi/article/4246) table S1. A cumulative dose measure was constructed by summing annual exposures.

### Record linkage with ICES (formerly known as Institute for Clinical Evaluative Sciences)

ICES is an independent, non-profit research institute whose legal status under Ontario’s health information privacy law allows it to collect and analyze healthcare and demographic data without consent for health system evaluation and improvement. The ICES data repository includes individual-coded data and longitudinal follow-up of health records that extend back to 1986 for almost 21 million people. The repository integrates health records of all residents eligible for healthcare in Ontario and includes physician claims, discharge summaries of hospital admissions, emergency department visits, and records of home and long-term care.

**Figure 1:**
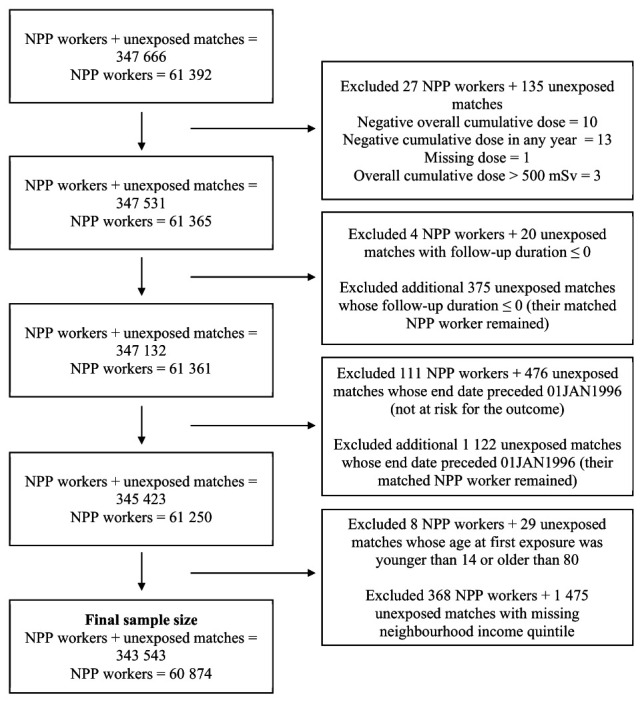
Flow diagram illustrating the study population. [NPP=nuclear power plant; mSv=millisieverts.]

The data file was restricted to individuals employed in a radiation-exposed occupation in Ontario from 1991 through 2022. The cohort was further restricted to those who had ever worked in an NPP. Once NPP workers were identified, we retrieved any previous (ie, before 1991) occupational dose records to account for exposure in previously monitored jobs. Additionally, we excluded all individuals in the NDR who had a record of being employed by Atomic Energy of Canada Limited. These individuals (N=3181) were excluded due to concerns about exposure measurement errors for doses. The finalized dataset was linked to Ontario health data housed at ICES with probabilistic record linkage using full names, birthdates, place of birth, and biological sex. These datasets were linked using unique encoded identifiers and analyzed at ICES. The flow diagram illustrating the creation of the final study population is shown in figure 1.

Neighborhood-level income quintiles were used as a proxy for socioeconomic status. ICES derived these quintiles by linking postal codes to census geography at the dissemination area level. Dissemination areas are the smallest areas in which population characteristics from the Canadian Census are reported. These areas are fairly stable and typically capture an overall population of 400–700 persons (29). Each dissemination area was assigned to an income quintile based on the distribution of household income from the most recent census.

### Measures of incident dementia

Incident dementias in the cohort of workers were identified using the Ontario Dementia Database, which employed an algorithm developed by ICES based on hospitalizations, physician visits, and prescription drug data (30, 31). This case definition has been previously applied in other cohorts (32, 33).

The Ontario Dementia Database was established in 1991 and includes a 5-year washout period to account for undiagnosed prevalent cases; thus, we were able to identify incident dementia beginning in 1996. The development and validation of this algorithm have been described elsewhere (31). Briefly, incident cases of dementia were defined as any individual aged 40–110 years old meeting one of the following criteria: ≥3 provincial hospital and physician record claims with a recorded dementia diagnosis [eg, Alzheimer’s disease (G30), unspecified dementia (F03), vascular dementia (F01)] according to the International Classification of Diseases (ICD) ninth and tenth editions (ICD-9: 290.0, 290.1, 290.2, 290.3, 290.4, 294.x, 331.0, 331.1, 331.5, 331.82; ICD-10: F00.x, F01.x, F02.x, F03.x, G30.x), which were each ≥30 days apart over two years; or ≥1 hospitalization same-day surgery with a dementia diagnosis recorded; or ≥1 prescription drug claim with a dementia medication (eg, donepezil, galantamine) dispensed. The dataset was created by combining these data sources with demographic information for persons eligible for healthcare coverage in Ontario. These data sources provide complete coverage of healthcare interactions incurred by Ontario residents. These are further described in supplementary table S2. This information was also used as a proxy for emigration from the province of Ontario when accruing person-years of follow-up and censoring individuals when they moved out of the province. Follow-up extended from 1996 onwards to the earliest of death, diagnosis of dementia, or the end of the study period (31 December 2022).

The Ontario Dementia Database has been found to identify individuals (aged ≥65 years) with Alzheimer’s and related dementias with a sensitivity of 79.3% and specificity of 99.1% (positive and negative predictive values of 80% and 99%, respectively) (31). Although the validation was performed among older adults (≥65 years), these findings are likely generalizable to those >45 years, as demonstrated by the findings of another validation study examining early-onset dementia (30). Specifically, among individuals aged 45–65 years, the algorithm identified Alzheimer’s and related dementias with a sensitivity of 72.9% and specificity of 99.7% (30).

### Statistical analysis

Descriptive analyses were performed to summarize key characteristics of the cohort, including age and sex frequencies and the exposure distribution.

Internal cohort comparisons were conducted to assess the exposure–response relationship between radiation and incident dementia. The shape of the exposure–response relationship was assessed using categories of cumulative exposure in mSv (0, 0.01–0.99, 1–4.99, 5–9.99, 10–24.99, 25–49.99, 50–99.99). Individuals with cumulative lifetime exposures exceeding 100 mSv were excluded (N=1456); however, their person-time was accrued until they exceeded 100 mSv of cumulative exposure (person-years >100 mSv=21 775; 44 cases excluded). Additionally, sensitivity analyses were performed to assess the exposure–response relationship between radiation and incident dementia among workers with lifetime cumulative exposures <500 mSv (supplementary tables S3 and S4, figure S1). The statistical analysis used Poisson regression models adjusted for sex, neighborhood income quintile, attained age (five-year groups), and calendar period (six-year groups). Poisson regression is a common analytic approach used in occupational cohorts as it is capable of accounting for time-dependent changes in exposure. In addition to these categorical analyses, a 2-knot natural cubic spline analysis was used to evaluate non-linear relationships between cumulative dose and dementia. Additionally, cumulative exposures were lagged by 10 years to allow for a latent period between radiation exposure and dementia onset. This approach is commonly used in studies of solid cancers and other chronic conditions such as dementia (12, 15–17). Sensitivity analyses were conducted for alternative lag periods (0 and 5 years) and are provided in supplementary tables S5 and S6. Overdispersion was evaluated using the ratio of Pearson’s 𝜒^2^ statistic to its degrees of freedom, with no evidence of overdispersion detected (ratio=1.18).

It is standard practice in radiation health effects to fit Poisson excess relative risk models (ERR) (2), and these models estimate the ERR based on internal comparisons of cumulative dose and dementia incidence. Effect modification on the linear scale by duration of exposure (≤1, 2–5, ≥6 years), age at first exposure (≤40, 41–50, ≥51 years), birth cohort (≤1940, 1941–1950, ≥1951), and time since last exposure (≤8, 9–15, ≥16 years) were also examined.

An external cohort analysis of incident dementia rates among Ontario NPP workers compared to the general Ontario population was conducted using a matched cohort design. Namely, the NPP worker cohort was matched to a random sample of the Ontario population by sex, age (2-year groups) and area of residence using a 5:1 sampling method. Matching by area of residence accounted for both regional differences in access to dementia care across Ontario (34) and regional variation in background radiation levels. For context, the average background radiation dose in Canada is 1.8 mSv per year (35); in contrast, some workers in our cohort accumulated annual occupational doses that substantially exceeded this background. Although this method differs from traditional indirect standardization, the use of relative risk (RR) between the two groups provides a comparable estimate to the standardized incidence ratio (SIR).

We conducted analyses using SAS 9.4 (SAS Institute, Cary, NIC, USA), and Epicure software to model linear excess RR (Hirosoft International, Eureka, CA, USA). Spline analysis was conducted using RStudio (RStudio Team, Boston, MA, USA).

## Results

Our study cohort comprised 60 874 NPP workers from the NDR employed in Ontario after 1991. The cohort was predominantly male (85.9%), and the average age at the start of follow-up was 36.2 years old [standard deviation (SD) 11.8, table 1]. These workers were followed for an average of 13.5 years (SD 8.6), accumulating 867 028 person-years at risk. Almost 40% of the cohort had zero exposure at the end of follow-up, and the mean cumulative dose at the end of follow-up was 11.7 mSv (SD 31.3, min=0, max=478.8, interquartile range 8.1). In total, there were 476 incident dementia cases identified. These summary statistics reflect the entire cohort without restricting to those <100 mSv, as they were calculated at the individual level rather than based on person-years.

**Table 1:** Descriptive characteristics for the nuclear power plant workers (N=60 874), 1996–2022. [mSv=millisievert; SD=standard deviation]

		N (%)	Mean (SD)
Sex			
	Male	52 307 (85.9)	
	Female	8567 (14.1)	
Age at start of follow-up (years)			
	≤30	22 796 (37.5)	
	30–35	8402 (13.8)	
	36–40	7902 (13.0)	
	41–45	7161 (11.8)	
	46–50	6030 (9.9)	
	51–55	4553 (7.5)	
	56–60	2747 (4.5)	
	61–65	994 (1.6)	
	66–70	236 (0.4)	
	≥71	53 (0.1)	
Neighbourhood income quintile			
	Q1 (Lowest)	8586 (14.1)	
	Q2	11 155 (18.3)	
	Q3	12 440 (20.4)	
	Q4	14 118 (23.2)	
	Q5 (Highest)	14 575 (23.9)	
Follow-up duration (years)			
	<1	2547 (4.2)	
	1–10	23 784 (39.1)	
	11–20	20 200 (33.2)	
	≥21	14 343 (23.6)	
Cumulative dose at the end of follow-up (mSv)			11.67 (31.3)
Age at first exposure (years)			33.96 (11.8)
Age at last exposure (years)			43.93 (13.4)
Birth year			1971.46 (15.4)
Age at the start of follow-up (years)			36.17 (11.8)
Follow-up duration (years)			13.48 (8.6)
Year of dementia diagnosis			2015.74 (4.7)
Age at diagnosis (years)			70.53 (8.7)

Findings from the internal cohort analyses are presented in table 2. In the adjusted model, there was a positive statistically significant (P=0.022) dose–response relationship between cumulative exposure to whole-body, low-dose radiation and the risk of dementia. Specifically, the RR of dementia in the lowest exposure category (0.01–0.99 mSv) was 1.63 (95% CI 1.26–2.12) compared to the zero-dose category, and the RR in the highest exposure category (50–99.99 mSv) was 1.50 (95% CI 0.99–2.28) compared to the zero-dose category. The excess risk per 100 mSv from fitting a linear excess RR model was 0.704 (95% CI 0.018–1.390). When workers with lifetime cumulative exposures <500 mSv were considered, the linear excess RR decreased but remained statistically significant [ERR/100 mSv=0.238 (95% CI 0.0024–0.474)] (table S3).

**Table 2:** Adjusted relative risks (RR) and linear excess relative risk [ERR/100 millisieverts (mSv)] for incident dementia, lagged 10 years, Ontario nuclear power plant workers, lifetime cumulative exposure <100 mSv, 1996–2022.^1^ [CI=confidence interval]

		Mean (SD)	N (%)	Person-years (%)	RR (95% CI)	P-value for trend
Category						0.0217
	0	0.00	160 (37.0)	586 757 (67.7)	Reference	
	0.01–0.99	0.34 (0.3)	89 (20.6)	79 321 (9.2)	1.63 (1.26–2.12)	
	1–4.99	2.57 (1.2)	56 (13.0)	61 155 (7.1)	1.51 (1.11–2.06)	
	5–9.99	7.25 (1.4)	26 (6.0)	32 409 (3.7)	1.50 (0.99–2.28)	
	10–24.99	16.46 (4.3)	32 (7.4)	48 502 (5.6)	1.24 (0.85–1.82)	
	25–49.99	35.58 (7.1)	43 (10.0)	34 095 (3.9)	2.13 (1.52–3.00)	
	50–99.99	70.40 (14.4)	26 (6.0)	24 789 (2.9)	1.50 (0.99–2.28)	
RR (quasi-continuous)			432 (100)	867 028 (100)	1.005 (1.00–1.01)	
ERR/100 mSv			432 (100)	867 028 (100)	0.704 (0.018–1.390)	

^1^ Adjusted for sex, neighborhood income quintile, attained age, and calendar period.

The results from the evaluation of various effect modifiers are summarized in Table 3. We observed some indication of a dose-rate amplification effect (P=0.01), with workers exposed for ≤1 year showing a higher excess risk (ERR/100 mSv=10.25; 95% CI -3.74–24.24) compared to those with longer exposure durations (≥6 years, ERR/100 mSv=0.49; 95% CI -0.19–1.17). However, given the high degree of uncertainty, these results must be interpreted with caution. There was no clear pattern of effect modification by time since last exposure, and trends by birth cohort and age at first exposure were not statistically significant (P=0.20 and P=0.26, respectively).

**Table 3:** Effect modification in the linear scale using excess relative risk [ERR/100 millisieverts (mSv)], lagged 10 years, Ontario nuclear power plant workers, lifetime cumulative exposure <100 mSv, 1996–2022.^1^ [CI=confidence interval; LRT=likelihood ratio test]

		N (%)	Person-years (%)	ERR/100 mSv (95% CI)	LRT p-value
Overall		432 (100)	867 028 (100)	0.704 (0.018–1.390)	
Duration of exposure (years)					0.01
	≤1	232 (53.7)	655 509 (75.6)	10.25 (-3.74–24.24)	
	2–5	108 (25.0)	110 899 (12.8)	3.54 (0.77–6.31)	
	≥6	92 (21.3)	100 620 (11.6)	0.49 (-0.19–1.17)	
Time since last exposure (years)					> 0.5
	≤8	165 (38.2)	741 006 (85.5)	0.39 (-0.54–1.31)	
	9–15	157 (36.3)	89 297 (10.3)	1.26 (-0.0004–2.51)	
	≥16	110 (25.5)	36 725 (4.2)	0.72 (-0.53–1.98)	
Birth cohort					0.20
	≤1940	120 (27.8)	14 084 (1.6)	1.82 (0.20–3.43)	
	1941–1950	194 (44.9)	94 422 (10.9)	0.41 (-0.47–1.29)	
	≥1951	118 (27.3)	758 522 (87.5)	0.26 (-0.84–1.37)	
Age at first exposure (years)					0.26
	≤40	156 (36.1)	641 812 (74.0)	0.61 (-0.11–1.34)	
	41–50	103 (23.8)	145 503 (16.8)	0.80 (-0.80–2.40)	
	≥51	173 (40.0)	79 713 (9.2)	3.92 (-0.51–8.35)	

^1^ Adjusted for sex, neighborhood income quintile, attained age, and calendar period.

**Figure 2:**
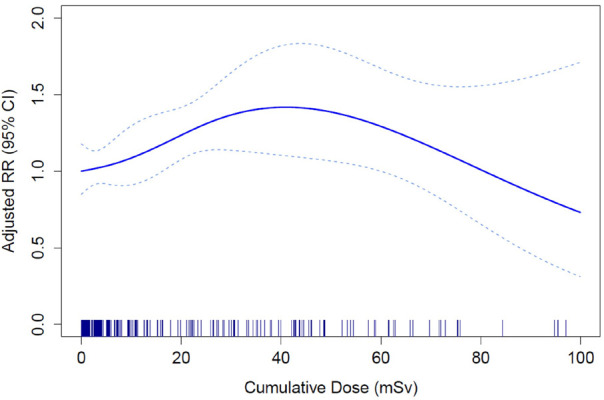
Attained age-, sex-, neighborhood income quintile-, and calendar period-adjusted relative risk (RR) (solid line) and 95% confidence intervals (CI) (dotted lines) for increasing radiation exposure (10-year lag) and dementia risk by 2-knot natural cubic spline model.

Internal cohort analysis using 2-knot natural cubic splines suggests that the shape of the dose–response curve is inconsistent with a linear relationship (on a log scale) between cumulative dose and dementia (figure 2). The curve showed increased risks of dementia at lower cumulative doses. At higher doses, the risk appears to decrease; however, the wide CI throughout indicate considerable uncertainty.

Findings from the external cohort comparison indicate that NPP workers had a 20% reduced risk of dementia (SIR 0.80; 95% CI 0.72–0.89) compared to the Ontario general population.

## Discussion

This study investigated the association between occupational exposure to low-dose ionizing radiation and dementia incidence in a cohort of Ontario NPP workers. Our internal cohort analyses revealed elevated dementia risks among exposed workers, and the linear excess RR model was suggestive of a dose-response trend. We observed that NPP workers had a significantly reduced risk of dementia compared to the general population of Ontario. This reduced risk is likely a result of the healthy worker effect, including healthy hire and healthy survivor effects, where employed populations typically have better health outcomes than the general population due to selective workforce entry and ongoing employment (36). Furthermore, NPP workers may differ from the general population in terms of education level or workplace cognitive demands. Lower education in early life has been identified as a risk factor for developing dementia (37). Workers in nuclear facilities must undergo rigorous training and certification requirements set by the CNSC, which may result in a cohort with higher baseline cognitive abilities than the general Ontario population. While we adjusted for neighborhood income quintile to partially account for socioeconomic differences, there may be residual confounding due to unmeasured factors such as individual-level education and job-related cognitive demands.

Our findings differ from those reported by Yamada et al (22), whose findings were not statistically significant, but suggestive of an inverse association between radiation and incident dementia, where diagnoses were based on cognitive tests and DSM-IV criteria. Specifically, they reported a hazard ratio of 0.82 (95% CI 0.59–1.14) among atomic bomb survivors exposed to radiation <500 mGy compared to doses <5 mGy (1 mGy ≈ 1 mSv for gamma radiation). However, as described earlier, our study is not directly comparable as the survivors of the atomic bombings were exposed to acute high-dose radiation, whereas we were focused on chronic low-dose radiation exposures. A study by Ivanov et al (38) reported statistically significant increases in the incidence of the ICD category “Mental and Behavioral Disorders” among Chernobyl liquidators. In contrast, Rahu et al (39) found little evidence of increased risks in the same category among Estonian Chernobyl cleanup workers compared to the general Estonian population. However, the findings (38, 39) are limited, as they examined broad outcome categories in which dementia likely represents only a small subset.

Likewise, most studies examining the risk of dementia from radiation exposure have relied on mortality outcomes based on broad ICD categories, such as ‘Mental and Behavioral Disorders’ (11–17, 19, 21) or a combined category of ‘Dementia, Alzheimer’s Disease, Parkinson’s, and other motor neuron diseases’ (14–16, 21), often yielding mixed results. The lack of specificity in these categories increases the potential for outcome misclassification and may obscure more nuanced associations. Conversely, some studies (11, 12, 14–16, 18, 20, 21) have specifically examined mortality risks of ‘Dementia and Alzheimer’s disease’ and have also reported mixed findings. Additionally, dementia mortality studies frequently rely on external cohort analyses to compute standardized mortality ratios (SMR) (12–16, 18, 21). While SMR provide useful insights and help contextualize findings to the broader population, they may introduce biases due to differences in population characteristics. Internal cohort analyses are methodologically stronger because they allow for better control of confounders and reduce susceptibility to the healthy worker effects. An example of the healthy worker effect is seen in a study of dementia mortality by Boice et al (16), which found increased risks of the combined category of ‘Dementia, Alzheimer’s Disease, Parkinson’s, and other motor neuron diseases’ in their internal cohort analyses, while the comparison to the general US population showed decreased risks. Although internal and external comparisons differ methodologically, the contradictory findings in our study, in our view, highlight the need to rely on internal cohort comparisons for occupational studies of dementia.

Our analysis focuses on low-dose exposures, restricting to individuals with lifetime cumulative doses <100 mSv (mean 11.7 mSv), whereas most other studies have examined populations with substantially higher exposures. For instance, the atomic bomb survivor cohort (22) received an average radiation dose of 417.2 mGy (1 mGy ≈ 1 mSv for gamma radiation), which is considerably higher than in our study. Comparing cumulative doses without considering dose rates may not fully capture exposure differences, as a single dose of 417 mGy may have different health effects than 12 mSv accumulated over several years.

Identifying dementia in administrative health data presents challenges, as a large portion of cases are underdiagnosed and may be missing from routine administrative datasets (40). Many individuals with dementia visit hospitals for other reasons, leading to the underrepresentation of those with dementia when using hospital records where the most responsible diagnosis for treatment is relied upon. Complicating matters further, those with comorbidities may also have more frequent interactions with the healthcare system, increasing the likelihood of dementia detection. Furthermore, there are patient and physician characteristics that contribute to underdiagnosis, such as lack of education about dementia care, potential stigmatizing effects of diagnosis, and concerns about the consequences of misdiagnosing dementia (41). However, in our view, these factors are unlikely to be associated with radiation exposure. There are also challenges in the diagnostic criteria with the added complication of mild cognitive impairment, which is a condition that encompasses individuals who experience a cognitive decline from the past but function independently in their daily lives (42). While our available data do not allow for the distinction between dementia subtypes or mild cognitive impairment, the high sensitivity and specificity of the Ontario Dementia Database provide reassurance in accurately identifying cases. This validated algorithm draws on multiple data sources, including hospitalizations, insurance claims, and prescription data, enhancing the accuracy and comprehensiveness of dementia case identification. Further research is needed to investigate associations between low-dose ionizing radiation and specific dementia subtypes.

A limitation of our study was the lack of individual-level dementia risk factor information. Our data were derived from large administrative sources such as the Canadian National Dose Registry and Ontario ICES health data and lacked information on lifestyle-related behaviors identified as risk factors for dementia. These factors are varied and include cigarette smoking, alcohol use, diet, obesity, physical activity, education, and other sociodemographic factors (37). While these factors are unlikely to be differentially associated with radiation exposure within the cohort, they may still contribute to confounding through indirect relationships involving multiple characteristics. It is reassuring, however, that adjustment for neighborhood income quintile did not materially change our risk estimates.

Sex-based differences in radiation sensitivity have been suggested with potential biological mechanisms involving hormonal regulation and genetic factors (2, 43, 44). Women may be more vulnerable to the health impacts of ionizing radiation due to their greater proportion of radiation-sensitive reproductive tissues and differences in body size, which can influence the absorbed dose (2, 43). However, our cohort was predominantly male (86%), precluding meaningful analyses of sex-based differences in risk. Future research should consider other sectors in the NDR, such as medical professions, with a more balanced sex distribution and consider additional sociodemographic factors that may influence dementia risk.

Despite these limitations, our study has notable strengths. The Canadian NDR, of which approximately 16% are NPP workers, is one of the largest occupational radiation-exposed cohorts in the world, with extensive follow-up periods. This allows for evaluating long-term health outcomes, such as dementia, with individual-level exposure measures. Our study addresses key gaps in the literature by focusing on incident dementia and employing a validated algorithm to identify cases accurately. Unlike mortality-based studies, which overlook non-fatal cases and are affected by cause-of-death misclassification, incidence data offer a more comprehensive measure of disease occurrence. By using specific outcome definitions rather than broader ICD categories, we provide a clearer evaluation of potential radiation-related dementia risks.

This is the first study to report on the risk of occupational low-dose radiation exposure and dementia incidence. Our findings suggest that low levels of exposure may increase the risk of dementia. The statistically significant dose–response relationship and evidence of a non-linear association from spline analysis highlight the complexity of this relationship. These results emphasize the need for further research to better understand potential thresholds, mechanisms, and long-term cognitive effects of low-dose radiation exposure.

## Supplementary material

Supplementary material
