# Supplementary material for: Exposure to protracted low-dose ionizing radiation and incident dementia in a cohort of Ontario nuclear power plant workers
Source: Scand J Work Environ Health. 2025 Oct 30;51(6):495–504. doi: 10.5271/sjweh.4246 (PMC12591044; doi:10.5271/sjweh.4246)
Supplement: Supplementary material [file SJWEH-51-495-S001.pdf]

Exposure to protracted low-dose ionizing radiation and incident dementia in a cohort of Ontario nuclear power plant workers[1]

by Brianna Frangione, BSc,[2] Ian Colman, PhD, Franco Momoli, PhD, Estelle Davesne, PhD, Robert Talarico, MSc, Chengchun Yu, MSc, Paul J Villeneuve, PhD

1. Supplementary Material
2. Correspondence to: Brianna Frangione, PhD Candidate, Department of Neuroscience, Faculty of Science, Carleton University, Health Sciences Building, Room 2304, 1125 Colonel By Drive, Ottawa, ON K1S 5B6, Canada. [E-mail: brianna.frangione@carleton.ca]

Table S1. Breakdown of the types of radiation used in the overall dose calculation.

**Dose Types**	
Gamma/Beta (Whole Body)	Iron 59
Tritium Oxide	Cobalt 60
Tritium Oxide CED (Annual)	Carbon 14 Particulate
Radon Progeny	Other Fission/Activation Products (N<92)
Neutron (Fast)	Strontium 90
Neutron (Thermal)	Cesium 137
Long-lived Radioactive Dust	Technetium 99m
Cerium 144	Uranium Natural (U238, U234, U235)
Tritium Gas	Carbon 14 Dioxide
Iodine 131	Transuranic (N>92)
Iodine 125	Zirconium/Niobium 95
Radon Gas	Uranium Lung Burden
Americium 241	Uranium Urinalysis

Table S2. Data sources used in the case definition of dementia in the ICES-Derived Dementia Cohort. The dataset was created by combining these data sources with demographic information for persons eligible for health care coverage in Ontario (from the Registered Persons Database). The Ontario Dementia Database algorithm incorporates a 5-year washout period to ensure only incident cases of dementia are captured; thus, time-at-risk started in 1996.

Database	Period
Ontario Health Insurance Plan (OHIP)	July 1, 1991 to March 31, 2023
Canadian Institute for Health Information – Discharge Abstract Database (CIHI – DAD)	April 1, 1988 to March 31, 2023
Canadian Institute for Health Information – Same Day Surgery database (CIHI – SDS)	April 1, 1991 to March 31, 2023
Ontario Drug Benefit Database (ODB)	April 1, 1991 to March 31, 2023

Table S3. Adjusted relative risks (RR) and linear excess relative risk (ERR/100 mSv) for incident dementia, lagged 10 years, Ontario nuclear power plant (NPP) workers, lifetime cumulative exposure below 500 mSv, 1996 – 2022.

Cumulative dose category (mSv)	Mean (SD) cumulative dose (mSv)	N (%)	Person-years (%)	RR (95% CI)
0	0.00	160 (33.6)	586 757 (66.0)	Reference
0.01 – 0.99	0.34 (0.3)	89 (18.7)	79 321 (8.9)	1.64 (1.26–2.13)
1 – 4.99	2.57 (1.2)	56 (11.8)	61 155 (6.9)	1.51 (1.11–2.06)
5 – 9.99	7.25 (1.4)	26 (5.5)	32 409 (3.7)	1.50 (0.99–2.27)
10 – 24.99	16.46 (4.3)	32 (6.7)	48 502 (5.5)	1.24 (0.84–1.81)
25 – 49.99	35.58 (7.1)	43 (9.0)	34 095 (3.8)	2.13 (1.52–2.99)
50 – 99.99	70.40 (14.4)	26 (5.5)	24 789 (2.8)	1.50 (0.99–2.28)
100 – 499.99	180.80 (71.4)	44 (9.2)	21 775 (2.5)	1.69 (1.20–2.37)
P-value for trend				0.027
RR (Quasi-continuous)		476 (100)	888 803 (100)	1.002 (1.00–1.004)
ERR/100 mSv		476 (100)	888 803 (100)	0.238 (0.0024–0.474)

mSv: millisievert; CI: confidence interval

Adjusted for sex, neighbourhood income quintile, attained age, and calendar period

Table S4. Effect modification in the linear scale using ERR/100 mSv, lagged 10 years, Ontario nuclear power plant (NPP) workers, lifetime cumulative exposure below 500 mSv, 1996 – 2022.

	N (%)	Person-years (%)	ERR/100 mSv (95% CI)	LRT p-value
Overall	476 (100)	888 803 (100)	0.238 (0.0024–0.474)	
Duration of exposure (years)				0.01
≤ 1	232 (48.7)	655 509 (73.8)	9.71 (-3.91–23.32)	
2 – 5	108 (22.7)	110 937 (12.5)	3.15 (0.59–5.72)	
≥ 6	136 (28.6)	122 357 (13.8)	0.25 (0.004–0.49)	
Time since last exposure (years)				0.13
≤ 8	173 (36.3)	757 917 (85.3)	-0.054 (-0.36–0.26)	
9 – 15	176 (37.0)	92 609 (10.4)	0.49 (0.05–0.92)	
≥ 16	127 (26.7)	38 277 (4.3)	0.28 (-0.11–0.67)	
Birth cohort				0.38
≤ 1940	138 (29.0)	16 139 (1.8)	0.47 (0.02–0.93)	
1941 – 1950	213 (44.8)	102 427 (11.5)	0.11 (-0.18–0.40)	
≥ 1951	125 (26.2)	770 237 (86.7)	0.21 (-0.32–0.74)	
Age at first exposure (years)				0.20
≤ 40	198 (41.6)	663 366 (74.6)	0.24 (-0.006–0.48)	
41 – 50	105 (22.1)	145 724 (16.4)	0.40 (-0.52–1.33)	
≥ 51	173 (36.3)	79 713 (9.0)	3.62 (-0.64–7.88)	

mSv: millisievert; CI: confidence interval; LRT: likelihood ratio test

Adjusted for sex, neighbourhood income quintile, attained age, and calendar period

Table S5. Adjusted relative risks (RR) and linear excess relative risk (ERR/100 mSv) for incident dementia, 0- and 5-year lag periods, Ontario nuclear power plant (NPP) workers, lifetime cumulative exposure below 100 mSv, 1996 – 2022.

	0-year lag			5-year lag		
Cumulative dose category (mSv)	N	Person-years	RR (95% CI)	N	Person-years	RR (95% CI)
0	137	379 382	Reference	143	490 311	Reference
0.01 – 0.99	97	130 989	1.58 (1.22–2.05)	96	104 378	1.66 (1.28–2.15)
1 – 4.99	62	104 196	1.50 (1.11–2.02)	59	81 193	1.51 (1.11–2.05)
5 – 9.99	31	58 749	1.55 (1.04–2.29)	30	44 342	1.61 (1.08–2.39)
10 – 24.99	33	87 454	1.13 (0.77–1.66)	32	65 954	1.18 (0.80–1.73)
25 – 49.99	45	59 527	2.02 (1.44–2.84)	46	45 936	2.19 (1.56–3.06)
50 – 99.99	26	40 859	1.34 (0.88–2.04)	25	32 055	1.37 (0.89–2.10)
P-value for trend			0.13			0.07
RR (Quasi-continuous)	431	861 156	1.004 (1.00–1.01)	431	864 169	1.005 (1.00–1.01)
ERR/100 mSv	431	861 156	0.512 (-0.116–1.140)	431	864 169	0.593 (-0.059–1.25)

mSv: millisievert; CI: Confidence interval

Adjusted for sex, neighbourhood income quintile, attained age, and calendar period

Table S6. Effect modification in the linear scale using ERR/100 mSv, 0- and 5-year lag periods, Ontario nuclear power plant (NPP) workers, lifetime cumulative exposure below 100 mSv, 1996 – 2022.

	0-year lag				5-year lag			
	N	Person-years	ERR/100 mSv (95% CI)	LRT p-value	N	Person-years	ERR/100 mSv (95% CI)	LRT p-value
Overall	431	861 156	0.512 (-0.116–1.140)		431	864 169	0.593 (-0.059–1.25)	
Duration of exposure (years)				0.01				0.01
≤ 1	216	492 210	15.13 (-0.65–30.91)		222	579 556	15.48 (-0.53–31.50)	
2 – 5	114	183 793	2.89 (0.27–5.52)		108	144 353	2.74 (0.14–5.34)	
≥ 6	101	185 153	0.39 (-0.25–1.02)		101	140 260	0.47 (-0.19–1.12)	
Time since last exposure (years)				0.29				0.37
≤ 8	164	735 139	0.12 (-0.66–0.89)		164	738 152	0.21 (-0.62–1.04)	
9 – 15	157	89 292	1.24 (-0.01–2.49)		157	89 292	1.25 (-0.001–2.51)	
≥ 16	110	36 725	0.71 (-0.54–1.97)		110	36 725	0.72 (-0.54–1.97)	
Birth cohort				0.11				0.14
≤ 1940	120	14 047	1.81 (0.20–3.42)		120	14 056	1.82 (0.21–3.43)	
1941 – 1950	193	93 740	0.24 (-0.57–1.06)		193	94 027	0.29 (-0.54–1.13)	
≥ 1951	118	753 369	0.07 (-0.87–1.01)		118	756 086	0.15 (-0.86–1.16)	
Age at first exposure (years)				0.46				0.45
≤ 40	156	636 199	0.56 (-0.14–1.26)		156	639 042	0.62 (-0.10–1.34)	
40 – 50	102	145 249	0.05 (-1.16–1.26)		102	145 414	0.20 (-1.11–1.51)	
≥ 51	173	79 708	1.98 (-0.94–4.89)		173	79 713	2.38 (-0.91–5.67)	

mSv: millisievert; CI: confidence interval; LRT: likelihood ratio test

Adjusted for sex, neighbourhood income quintile, attained age, and calendar period

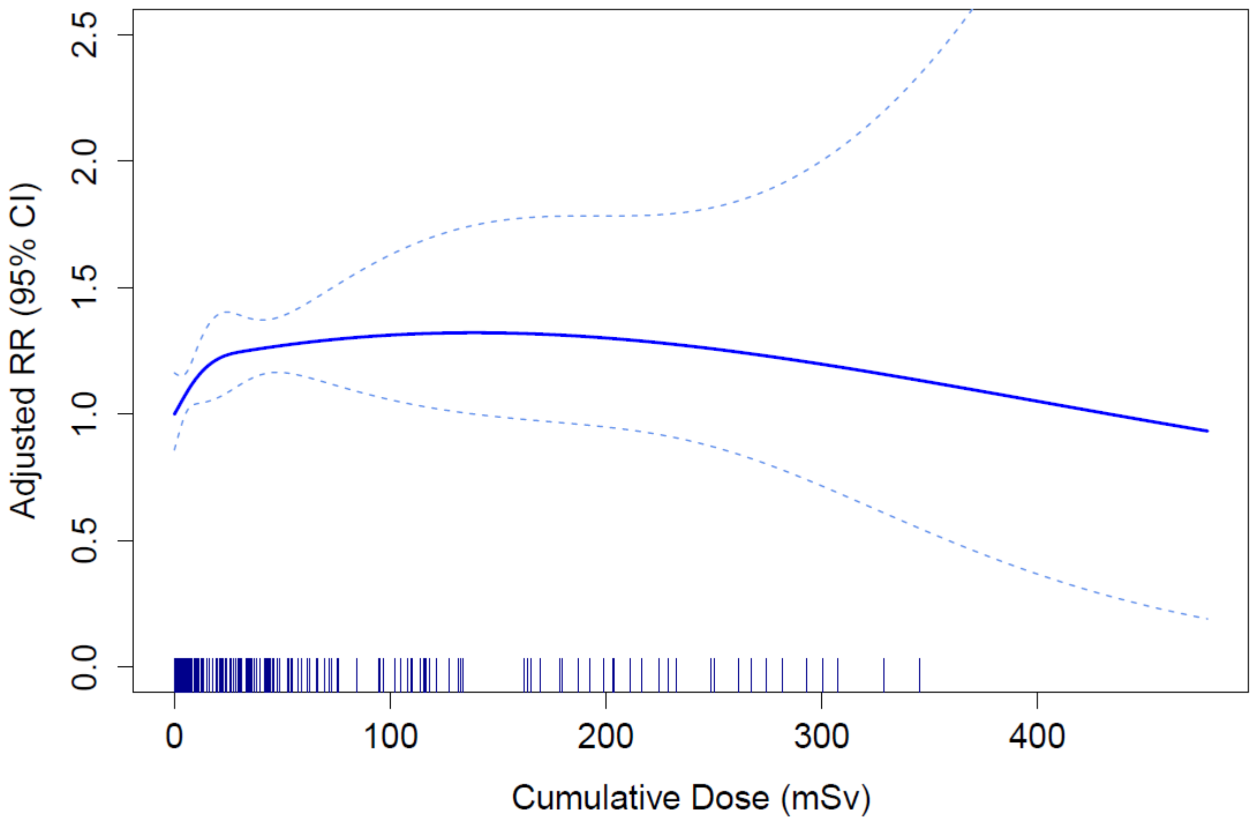

Figure S1. Attained age-, sex-, neighbourhood income quintile-, and calendar period-adjusted RR (solid line) and 95% confidence intervals (dotted lines) for increasing radiation exposure below 500 mSv cumulative exposure (10-year lag) and dementia risk by 2-knot natural cubic spline model.
